# Annotations

**Published:** 1911-03-25

**Authors:** 


### Annotations.

### Syphilis and "606."

When the unexampled and systematic booming of arsenobenzol for syphilis began in this country about six months ago, a note of warning—almost of incredulity—was sounded in these columns (The Hospital, August 20, 1910, p. 602). Many of our readers regarded the cautious non-committal tone of that pronouncement as pessimistic, or even old-fashioned. As usual however with artificial booms of all kinds, a reaction has set in, as we foretold; and a prominent syphilologist (Mr. C. F. Marshall) now sums up the present state of our knowledge in the following terse conclusions. There is no proof (he says) that an abortive cure of syphilis can be effected by "606" or any other drug. There is no evidence that parasyphilitic manifestations can be prevented by it. Arsenobenzol certainly has a rapid healing effect on certain syphilitic lesions, chiefly of the ulcerative type; but this is not constant, and is often only temporary. If arsenobenzol is indicated at all, it is chiefly in those cases which are not influenced by mercury; but such cases are rare, and moreover some of them resist both drugs. Arsenobenzol has numerous contra-indications. It appears to have little or no effect on visceral syphilis and on parasyphilitic affections. The injection, especially the intravenous injection, of the drug is attended with grave risks to the patient. In the present state of our knowledge there is no drug which can replace mercury in the treatment of syphilis. Mr. Marshall is, in fact, much more sceptical now after several months of actual experience, than was The Hospital at a time when only one or two men in England had ever seen this preparation of arsenic. Meanwhile a section of the public has been handsomely plundered for the benefit of a handful of enterprising but not too scrupulous specialists, and the rest of the profession has shown a degree of gullibility which would be astonishing could it not easily be paralleled from the experience of the past.

### The Latest Fad.

A few years ago it was eggs; then oranges; later still, sour milk; and now it is a particular brand of flour: all of these fads have had brief but prodigious booms, skilfully engineered by clever people with axes to grind, and each, so far, has sunk into that oblivion which doubtless awaits the latest panacea for every evil that afflicts mankind. In so far as the agitation is one which has a purely commercial basis, the whole episode has a ludicrous side which has not escaped the genial banter of that castigator of contemporary folly, *Mr. Punch*. In so far as it reflects the credulity and docility of the general public, it affords an instructive example of some of the most enduring traits of human nature and human weakness. But beyond all this there is an aspect of the affair which more especially affects the medical profession. For two or three weeks the painful spectacle has been exhibited to the world of a dozen or so well-known English medical men aiding and abetting by their signature a purely log-rolling exploitation of a commercial product. What the motives were that induced these gentlemen to append their names to the document in question we need not too closely inquire; suffice it that there are names to be found in the list which have hitherto not been associated with this sort of publicity-mongering. Of the result it is possible to speak much more emphatically. The ill-advised complaisance of these signatories has made not only themselves but British medicine the laughing-stock of Europe, indeed of the world. An unpleasant fact, but one which has got to be faced. When the usual fate of these ephemeral fads overtakes this latest craze, when the artificially-engineered boom dies away and sober reflection causes thinking people to analyse the ingredients of which it has been compounded, we do not envy the "eminent doctors" their reflections.

### The Doctor's Top Hat.

Not so very long since, even in rural districts, the medical man was easily recognisable to the stranger by his professional top hat and frock coat. Scarcely fifteen years ago the doctor was almost as unmistakable as the clergyman. There were few then who dared to break away from the conventional costume decreed by unwritten professional law, and those who did defy this canon generally suffered for their hardihood. Except perhaps in the height of summer the silk hat was almost obligatory upon the doctor, as much in small country towns as in London and the large cities. But within the past dozen years a complete change has come over the country practitioner's costume; and the main cause of this revolution has been the advent of the motor-car. Except in larger towns the doctor's motor-car has killed the doctor's top-hat; and public and profession alike have accepted this change with equanimity. But there must be many old-fashioned country people who have experienced a real sense of loss in the passing of their doctor's professional uniform. And, indeed, with all its obvious faults, the top hat and its associated coat often seem to lend some additional dignity and impressiveness even to the most dignified and impressive members of our profession. The doctor above all men should pay great attention to his outward appearance—"for the apparel oft betrays the man." It is not humbug or charlatanry which impels him to look well-dressed and well-groomed on all occasions; and this effect was certainly easier to obtain in the old days before the ubiquitous motor-car swept the doctor's top hat into oblivion. To the young and inexperienced man the formal coat was often a definite help in acquiring the elusive "bedside manner," and in giving the best possible first impression to his patients and their friends. It is not actors alone who feel the advantage of being "dressed to the part." But the old order has changed, and perhaps in a few years the top hat will be a thing of the past even in the heart of Harley Street itself.

